# Supplementary material for: Content-rich biological network constructed by mining PubMed abstracts
Source: BMC Bioinformatics. 2004 Oct 8;5:147. doi: 10.1186/1471-2105-5-147 (PMC528731; doi:10.1186/1471-2105-5-147)
Supplement: Additional File 2 — The original results of the above study (non-essential files are deleted to keep the file size under the limit set by BMC bioinformatics). [file 1471-2105-5-147-S2.bz2 › chilibotAdditionalFile2/dip05/609ID10329167E10991/html/CBXLC_CBXSC.html]

 


 **CBXLC** and **CBXSC** 
  
Found 1 abstracts in PubMed, retrieved 1.  
 

 What does Google say? 
 PDF only 
| .edu only 

---

- Biochemistry, 1992   **A hybrid ribulosebisphosphate carboxylase oxygenase enzyme exhibiting a substantial increase in substrate specificity factor.** .
  Two hybrid ribulose bisphosphate carboxylase oxygenase RubisCO enzymes were constructed using RubisCO small subunit **[CBXSC]** genes rbcS from two eucaryotic marine organisms, Cylindrotheca sp.
  N1 and Olisthodiscus luteus, cloned downstream of the RubisCO large subunit **[CBXLC]** gene rbcL of the cyanobacterium Synechococcus PCC 6301.
  The expression products synthesized by Escherichia coli JM107 pVTAC223 and pANOLI were purified and examined by polyacrylamide gel electrophoresis and compared to the purified products generated by E. coli MV1190 pBGL710 , containing cyanobacterial rbcL and rbcS genes.
  Both Cylindrotheca and Olisthodiscus small subunits were able to assemble in vivo with the Synechococcus large subunit octamer to form heterologous hexadecameric L8S8 enzymes, the pVTAC223 and pANOLI hybrid enzymes, respectively.
  Like the Synechococcus RubisCO, the hybrid enzymes were rapidly activated by Mg2 plus HCO3 , even in the presence of RuBP.
  The hybrid enzymes were considerably more sensitive to the competitive inhibitor 6 phosphogluconate.
  Detailed kinetic analysis indicated that while the carboxylase activity of both chimeric enzymes was severely reduced, in the case of the pVTAC223 hybrid enzyme, the degree of partitioning between carboxylation and oxygenation was increased nearly 60% relative to the Synechococcus RubisCO.
  Other kinetic properties, including the Michaelis constants for the gaseous substrates and RuBP, were altered in the hybrid proteins.
  These studies also led to the finding that the substrate specificity factor of the Cylindrotheca RubisCO is unusually high.
